# Identification of prognostic hub genes and therapeutic targets for selenium deficiency in chicks model through transcriptome profiling

**DOI:** 10.1038/s41598-023-34955-6

**Published:** 2023-05-29

**Authors:** Lakshman Kumar Lingamgunta, Bindu Prasuna Aloor, Sreenivasulu Dasari, Ranjani Ramakrishnan, Mahendran Botlagunta, Ashok Kumar Madikonda, Shankar Gopal, Ankanna Sade

**Affiliations:** 1 Department of Biochemistry, Sri Venkateswara University, Tirupati, 517502 Andhra Pradesh India; 2 Department of Botany, Rayalaseema University, Kurnool, 518002 Andhra Pradesh India; 3 Department of Virology, Sri Venkateswara University, Tirupati, 517502 Andhra Pradesh India; 4 School of Biosciences, Engineering and Technology, Vellore Institute of Technology (VIT), Bhopal, 466114 Madhya Pradesh India; 5 Department of Biochemistry & Molecular Biology, Central University of Kerala, Periye, 671316 Kerala India; 6 Department of Botany, Sri Venkateswara University, Tirupati, 517502 Andhra Pradesh India

**Keywords:** Computational biology and bioinformatics, Diseases

## Abstract

Selenium deficiency is a prevalent micronutrient deficiency that poses a major health concern worldwide. This study aimed to shed light on the molecular mechanisms underlying selenium deficiency using a chick model. Chickens were divided into control and selenium deficient groups. Plasma samples were collected to measure selenium concentration and transcriptome analyse were performed on oviduct samples. The results showed that selenium deficiency led to a significant reduction in plasma selenium levels and altered the expression of 10,266 differentially expressed genes (DEGs). These DEGs primarily regulated signal transduction and cell motility. The molecular function includes GTPase regulatory activity, and KEGG pathway analysis showed that they were mainly involved in the signal transduction. By using Cytoscape and CancerGeneNet tool, we identified 8 modules and 10 hub genes (*FRK, JUN, PTPRC, ACTA2, MST1R, SDC4, SDC1, CXCL12, MX1* and *EZR*) associated with receptor tyrosine kinase pathway, Wnt and mTOR signaling pathways that may be closely related to cancer. These hub genes could be served as precise diagnostic and prognostic candidate biomarkers of selenium deficiency and potential targets for treatment strategies in both animals and humans. This study sheds light on the molecular basis of selenium deficiency and its potential impact on public health.

## Introduction

Selenium (Se) is an essential trace element that plays critical roles in various metabolic processes, reproduction, antioxidant defence, and immune system of animals and humans^1–3^. The first nutritional role of selenium (Se) was recognized in livestock species in 1950, which showed that Se prevented pathologies in vitamin E-deficient animals^4^. Several reports suggest that dietary selenium supplementation can prevent certain forms of chronic diseases, such as cardiovascular diseases, diabetes, and cancer^5^. Plasma Se content is considered a useful biomarker of both Se status and dietary intake, and deficient selenium status has been associated with increased risk of diseases such as cardiovascular diseases, cancer, and viral infections like COVID-19 (coronavirus disease 2019), and HIV (human immunodeficiency virus)^6,7^. Previous studies have demonstrated that selenium deficiency can lead to impaired growth, which in turn can result in the development of pancreatic atrophy, exudative diathesis, and muscular dystrophy^8^. These conditions are often linked together and are caused by various types of lesions^8^. Pancreatic atrophy causes degenerative changes in the pancreas, exudative diathesis results in increased capillary fragility and haemorrhage, while muscular dystrophy leads to muscle wasting and oxidative stress^9,10^. Furthermore, dietary Se deficiency has also been found to be deleterious effects on egg production, and egg weight in laying hens and turkeys^11^.

However, the avian oviduct is a complex tissue and it has a dynamic cellular activity during egg formation^12^. Earlier transcriptomic studies in the chicken oviduct identified several novel genes, such as solute carriers, ATPase, avian beta-defensins, and calbindin, which are actively involved in the supply of ions and minerals for eggshell formation^13^. Solute carrier genes are critical to the oviduct, are mainly located in the cell membrane and encode membrane transport proteins^13^. They transport electrolytes, glucose, and amino acids^13^. *ATPase* (P-type adenosine triphosphatase) family genes with several members were identified in the uterus of laying hens. For instance, the *ATP2A3* (ATPase sarcoplasmic/endoplasmic reticulum Ca^2+^ transporting 3), *ATP2B2* (ATPase plasma membrane Ca^2+^ transporting 2), and *ATP2C2* (ATPase secretory pathway Ca^2+^ transporting 2) genes participate in various cellular activities, including intracellular calcium pumping, calcium signalling pathways and calcium transport^14^. Yacoub et al.^15^ and Mageed et al.^16^ suggested that the *AvBD* (avian β-defensin) gene encodes avian beta defensins that possess antimicrobial activity in the chicken oviduct and are incorporated into the eggshell membrane. Recently, next-generation sequencing (NGS) technology revealed that several novel genes, *CEMIP* (cell migration inducing hyaluronidase 1)*, CA2* (carbonic anhydrase 2)*, ERE* (epiregulin)*, SDC3* (syndecan-3)*, SLC6A17* (solute carrier family 6 member 17)*, SLC13A5* (solute carrier family 13 member 5), *SPP1* (secreted phosphoprotein 1)*, OVAL* (ovalbumin)*, OTOP2* (otopetrin 2)*, POMC* (proopiomelanocortin)*, PENK* (proenkephalin)*, PTN* (pleiotrophin)*,* and *WNT11* (wnt family member 11) that are involved in biological pathways regulate ion transport, eggshell calcification, and eggshell formation in aged laying hens given a selenium-enriched yeast diet^17^. However, recent studies suggest that selenium may be important for health not only human oviduct but also fallopian tubes^18^.

**Figure 1:**
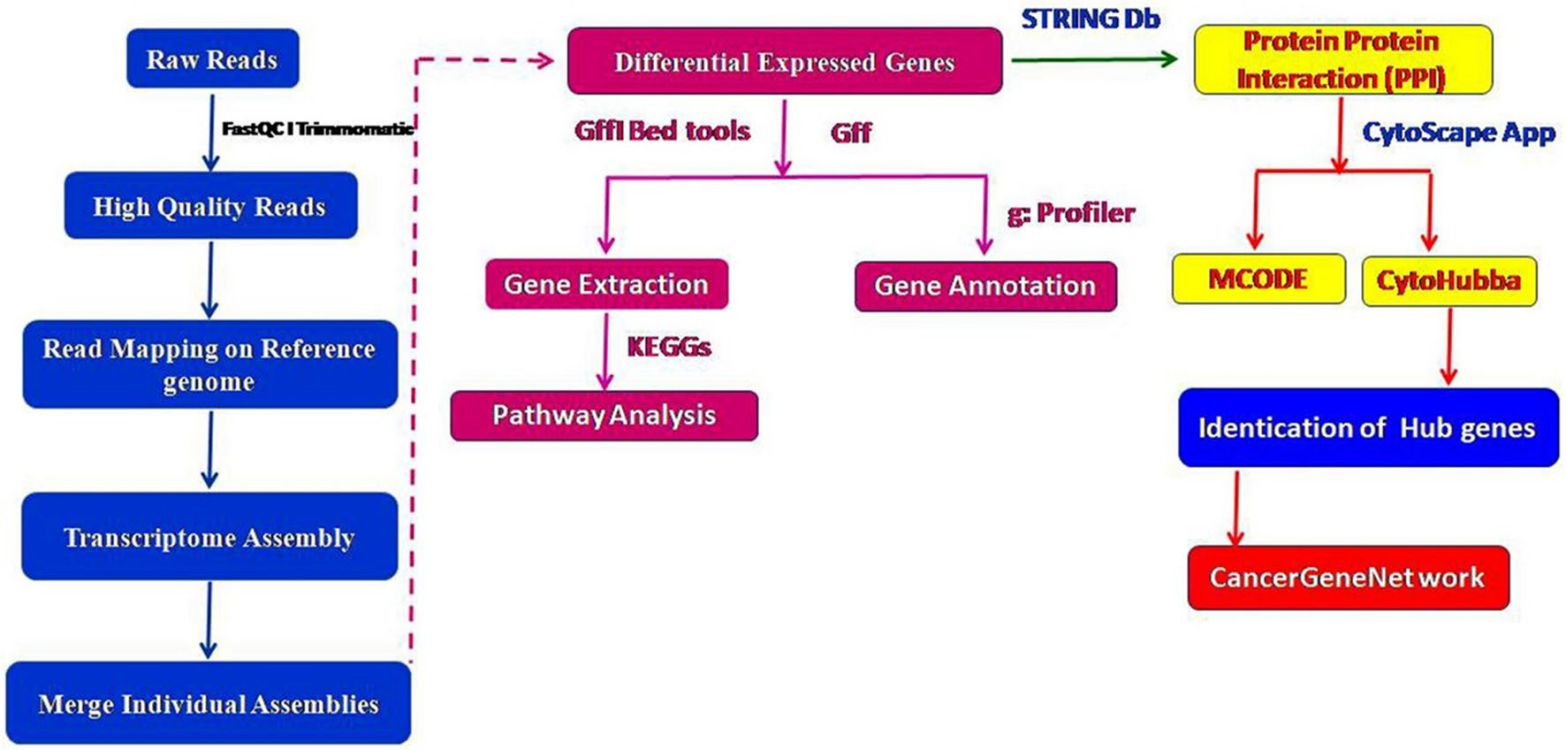
Outline of the bioinformatic workflow.

However, the molecular mechanisms of dietary selenium deficiency in the oviduct of chickens are poorly understood. Therefore, the present study was designed to investigate the relationship between Se status and gene expression profiles of the oviduct in a selenium-deficient chick model. In this regard, we measured Se status and identified selenium-deficient conditions in chicks. Next, we performed RNA sequencing (RNA-Seq)-based whole transcriptome analysis of a selenium-deficient versus control chicken oviduct to reveal novel key genes and biological pathways that regulate egg formation under selenium-deficient conditions. Differentially expressed genes (DEGs), heatmap, scatter plots, volcano plots, GO (gene ontology) characterization, and KEGG (Kyoto encyclopedia of genes and genomes) pathway analysis were used, and potential genes involved in selenium-deficiency disease conditions in the oviduct were analysed. Furthermore, we performed protein–protein interaction (PPI) network analyses of DEGs by the STRING database and then selected hub genes and modules of genes of the cluster of DEGs using the cytoscape software plugin with CytoHubba and Molecular Complex Detection (MCODE). Finally, we used CancerGeneNet network analysis of all hub genes. The brief bioinformatics workflow is depicted in Fig. 1. This study aimed to improve our understanding of the molecular mechanism and identify biomarker genes for selenium-deficiency conditions in a chick model and provide new treatment targets for selenium-deficiency disease treatment in humans.

## Results

### Selenium concentration in blood samples

**Figure 2:**
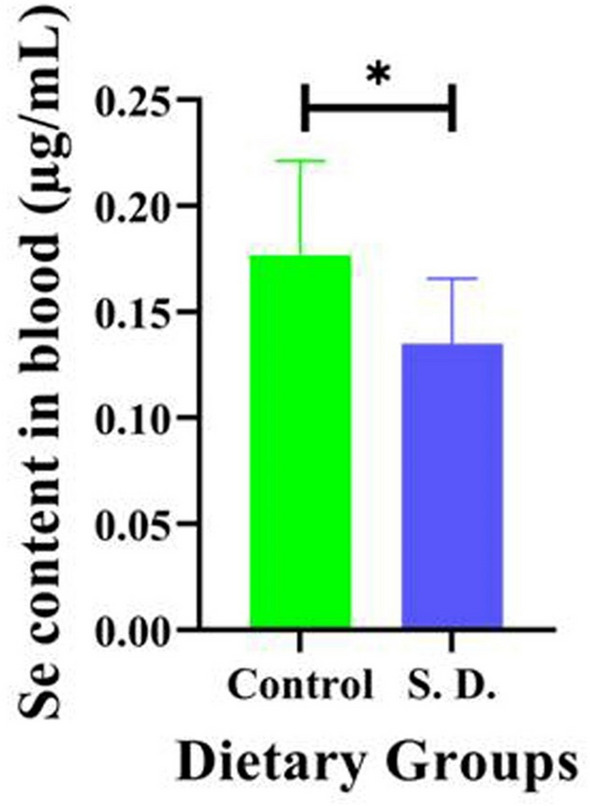
Selenium content in blood samples in the chickens.

In this study, chicks that were given a selenium-deficient diet showed a significant decrease in selenium concentration in their blood samples compared to chicks that were given a basal diet (*p* < 0.05), as shown in Fig. 2.

### Transcriptome data generation and processing

Illumina Next Seq500 technology was used to generate high-quality data for the control and selenium deficient groups, resulting in 3.35 and 2.14 Gb of data respectively. The sequencing process involved 2 × 150 bp chemistry. Tophat was used with default parameters to map the high-quality reads from each sample to the reference genome of *Gallus gallus* GRCg6a. The read mapping percentage was determined to be 75.6% and 77.6% for the control and selenium deficient groups, respectively. The details of these findings can be found in Supplementary Table S1 and Table S2.

### Identification of differentially expressed genes (DEGs)

Transcriptomes of individual oviduct tissues were assembled and analysed to identify differentially expressed genes (DEGs) between the selenium deficient and control groups using cufflinks and cuffdiff software. Significantly differentially expressed genes (DEGs) were identified based on statistical significance (*p* ≤ 0.05) and ≥ 2 log2 fold changes (FC) for upregulated genes, and ≤ − 2 log2 fold changes (FC) for downregulated genes. A total of 10,266 genes were differentially expressed in the control vs. selenium-deficient group. Of these, 213 genes were found to be significantly upregulated, and 237 were significantly downregulated. Supplementary Table S3 and Table S4 provide a complete list of the up- and downregulated differentially expressed genes (DEGs), while Supplementary Table S5 summarizes the differentially gene expression results. In addition, the raw data of differential expressed genes are provided in Supplementary Tables S6, S7, S8, S9 and S10.

**Figure 3:**
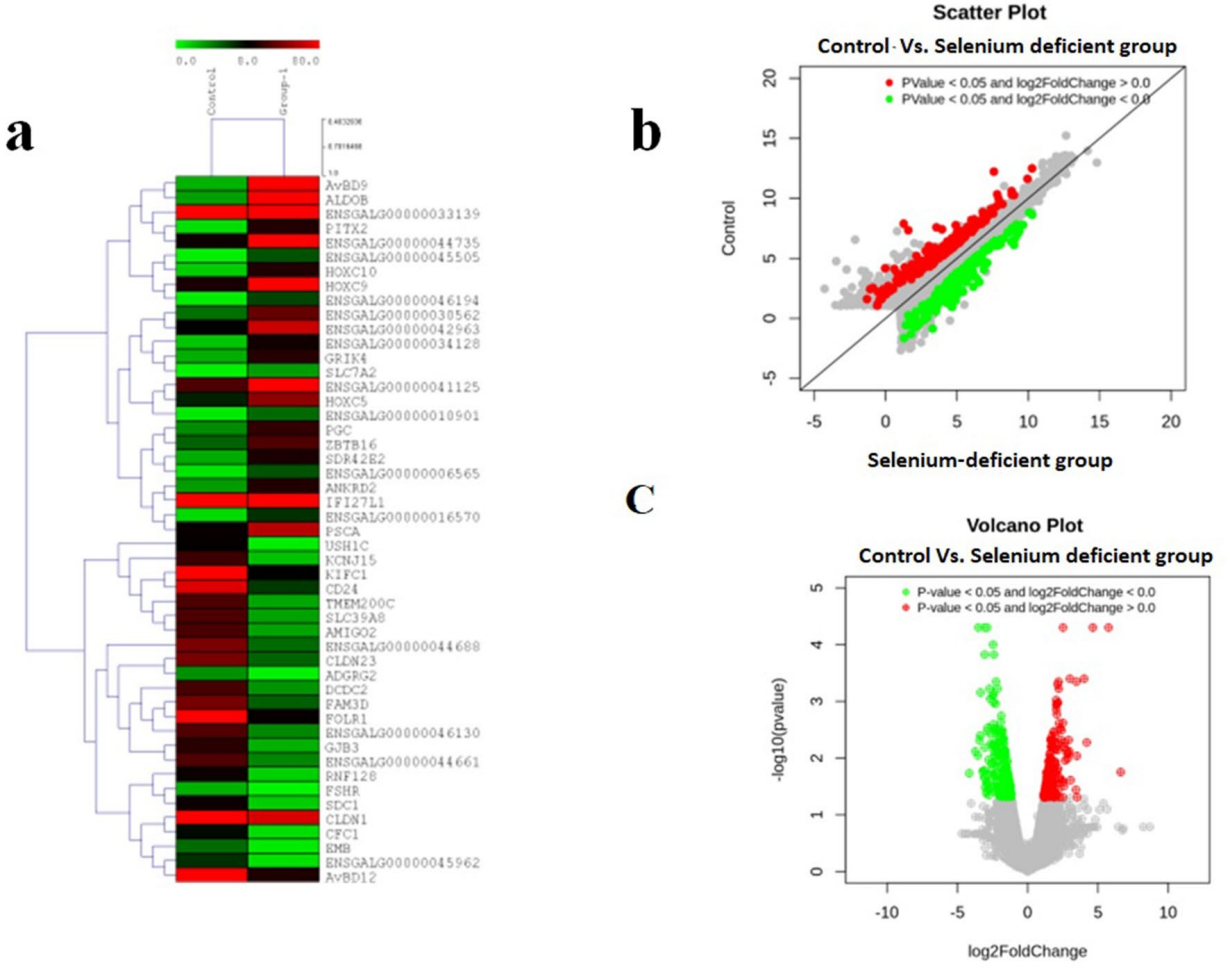
Visualization of differentially expressed genes (DEGs) using R studios (**a**) Heatmap of differentially expressed genes (DEGs) (Top 50) in the control versus selenium-deficient groups of the differential expression analysis. (**b**) Scatter plot of differentially expressed genes (DEGs); green dots represent the downregulated and red dots represent the upregulated genes in the control versus selenium-deficient diet groups. (**c**) Volcano plot of differentially expressed genes (DEGs); green dots represent the downregulated and red dots represent the upregulated genes in control versus selenium-deficient diet groups.

Heatmaps of differentially expressed genes (top 50) in the control vs. selenium-deficient group differential expression analysis are shown in Fig. 3a. The scatter plot of differentially expressed genes is shown; green dots represent the downregulated and red dots represent the upregulated genes in the control vs. selenium deficient groups (Fig. 3b). The volcano plot of differentially expressed genes is shown; green dots represent downregulated and red dots represent the upregulated genes in the control vs. selenium-deficient groups (Fig. 3c).

### Functional analysis of differentially expressed genes (DEGs)

**Figure 4:**
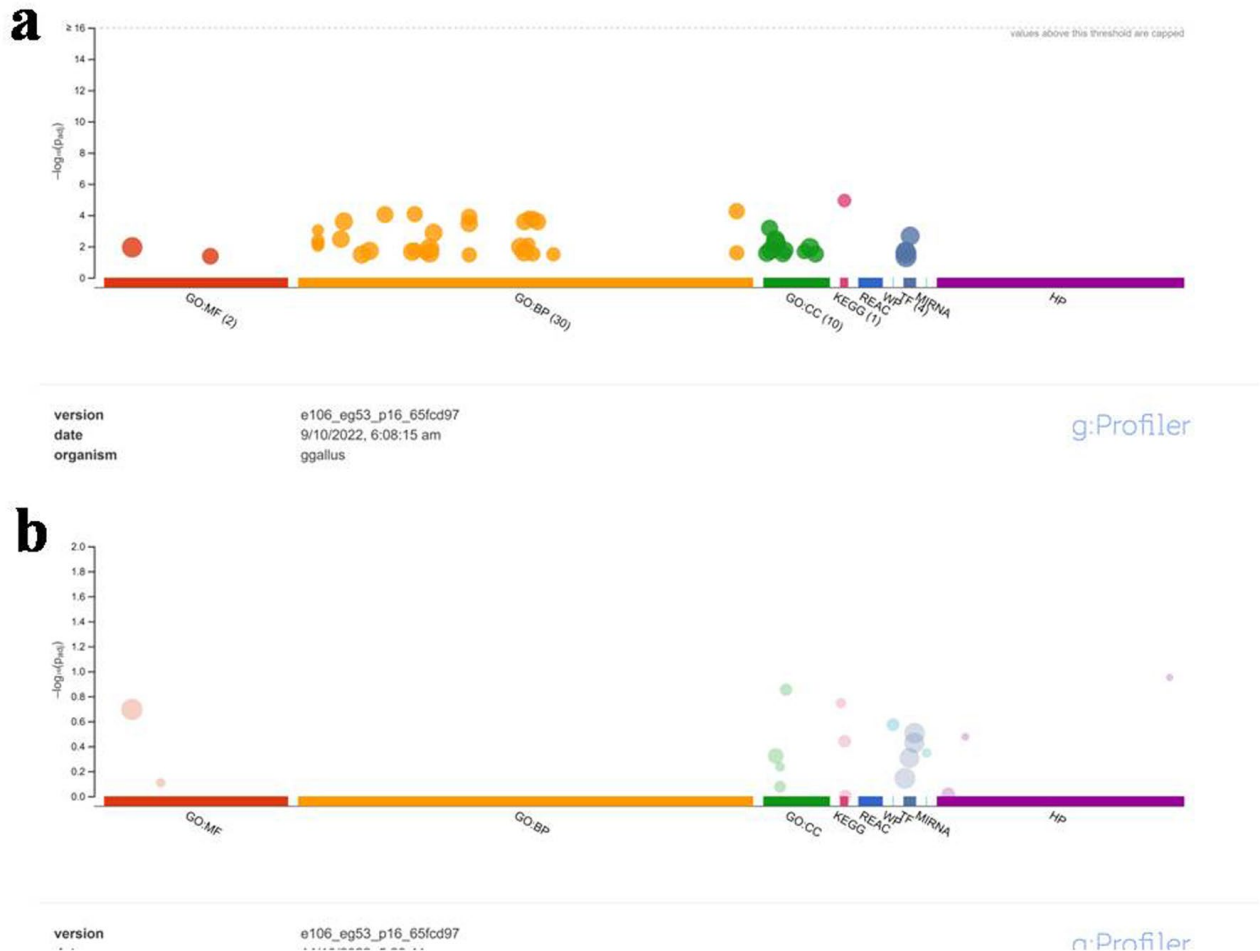
Gene Ontology (GO) enrichment analysis of differentially expressed genes (DEGs) in the oviduct of the control vs. selenium-deficient chickens. (**a**) biological process (BP), cellular component (CC), and molecular function (MF). The 194 upregulated DEGs were subjected to g: Profiler webserver for Gene Ontology (GO) enrichment analysis. The statistical parameter was used only for annotated genes. The significance threshold was the g: SCS threshold (*p* < 0.05). (**b**) biological process; BP, cellular components; CC, and molecular function; MF. The 146 downregulated DEGs were subjected to g: Profiler webserver for Gene Ontology (GO) enrichment analysis. The statistical parameter was used only for annotated genes. The significance threshold was the g: SCS threshold (*p* < 0.05).

The g: Profiler web server, a public functional annotation tool, was utilized to analyse the up and downregulated genes in chickens and gain insights into various Gene Ontology (GO) terms^19^. The official upregulated gene symbols were uploaded to the webserver, and *G. gallus* was chosen as the reference genome, resulting in 194 annotated genes across three GO terms: biological process (BP), cellular component (CC), and molecular function (MF) (Fig. 4a). Similarly, 146 downregulated genes were annotated across the three GO terms (Fig. 4b).

Further GO analysis was conducted on 213 upregulated and 237 downregulated differentially expressed genes (DEGs), assigning them to 90 enriched GO terms. The upregulated DEGs were annotated to 30 biological processes, two molecular functions, and 30 cellular components, with significant terms such as “cellular component movement,” “regulation of cell motility,” “regulation of signalling,” “regulation of cell migration,” “positive regulation of chemotaxis,” “regulation of signal transduction,” “regulation of locomotion,” and “cell communication.” Cellular component GO terms included “endoplasmic reticulum,” “endomembrane system,” “membrane,” and “vesicle.” Molecular functions enriched with “protein binding” and “protein-containing complex binding” were identified.

The downregulated differentially expressed genes (DEGs) were assigned to 30 BP (biological process), 30 MF (molecular function), and 30 CC (cellular component) Gene Ontology (GO) terms, with significant terms such as “cell surface receptor signalling pathway,” “signal transduction,” “cell adhesion,” “cell communication,” and “cell cycle.” The most relevant terms for molecular function were “binding,” “GTPase regulatory activity,” “inorganic cation transmembrane transporter activity,” and “carbohydrate binding.” Cellular component GO terms included “actin bundle filaments, “actin cytoskeleton,” and “phagocytic vesicle membrane.” All the identified Gene Ontology (GO) terms for both upregulated and downregulated genes are summarized in Tables 1 and 2, respectively.

**Table 1:** Gene Ontology (GO) terms for upregulated differentially expressed genes (DEGs).

Category	GO ID	GO term	Adjusted p-value
BP	GO:2000145	Regulation of cell motility	5.50E−05
BP	GO:0030334	Regulation of cell migration	8.49E−05
BP	GO:0050921	Positive regulation of chemotaxis	0.000132202
BP	GO:0009966	Regulation of signal transduction	0.032977079
BP	GO:0010646	Regulation of cell communication	0.01941158
BP	GO:0023051	Regulation of signalling	0.021871511
BP	GO:0040012	Regulation of locomotion	0.001326849
BP	GO:0051270	Regulation of cellular component movement	0.029940223
CC	GO:0005783	Endoplasmic reticulum	0.000678557
CC	GO:0012505	Endomembrane system	0.003640007
CC	GO:0016020	Membrane	0.005992789
CC	GO:0031982	Vesicle	0.01733706
MF	GO:0005515	Protein binding	0.011364633
MF	GO:0044877	Protein-containing complex binding	0.042467714

Note: ‘BP’, ‘CC’, and ‘MF’ represent biological process, cellular component and molecular function, respectively. Adjusted *p*-value.

**Table 2:** Gene Ontology (GO) terms for downregulated differentially expressed genes (DEGs).

Category	GO ID	GO term	Adjusted p-value
BP	GO:0007166	Cell surface receptor signalling pathway	1
BP	GO:0007165	Signal transduction	1
BP	GO:0007155	Cell adhesion	1
BP	GO:0007154	Cell communication	1
BP	GO:0007049	Cell cycle	1
BP	GO:0007259	Receptor signalling pathway via JAK-STAT	1
BP	GO:0040012	Cell–cell signalling	1
BP	GO:0007267	Ras protein signal transduction	1
CC	GO:0032432	Actin bundle filaments	0.140050699
CC	GO:0015629	Actin cytoskeleton	0.475423994
CC	GO:0030670	Phagocytic vesicle membrane	0.581274167
MF	GO:0005488	Binding	0.201762502
MF	GO:0031267	GTPase regulator activity	1
MF	GO:0022890	Inorganic cation transmembrane transporter activity	1
MF	GO:0030246	Carbohydrate binding	1

Note: ‘BP’, ‘CC’, and ‘MF’ represent biological process, cellular component and molecular function, respectively. Adjusted *p*-value.

Pathway enrichment analysis of up- and downregulated genes of differentially expressed genes (DEGs) in chickens was conducted using the Kyoto encyclopedia of genes and genomes (KEGG) database. The study identified 10, 266 differentially expressed genes (DEGs) in the control versus selenium deficient groups, which were classified into 23 functional pathway categories in the Kyoto encyclopedia of genes and genomes (KEGG) database. These pathways were further grouped into five specific categories in the Kyoto encyclopedia of genes and genomes (KEGG) database. These pathways were further grouped into five specific categories, namely, metabolism (A), genetic information processing (B), environmental information processing (C), cellular processes (D), and organismal system (E), as shown in Table 3.

**Table 3:** KEGG pathway classification.

Pathways^a^	Control vs selenium-deficient groups
	Gene counts
A. Metabolism	
Carbohydrate metabolism	208
Energy metabolism	147
Lipid metabolism	260
Nucleotide metabolism	113
Amino acid metabolism	185
Metabolism of other amino acids	67
Glycan biosynthesis and metabolism	184
Metabolism of cofactors and vitamins	135
Metabolism of terpenoids and polyketides	27
Biosynthesis of other secondary metabolites	28
Xenobiotics biodegradation and metabolism	69
B. Genetic information processing	
Transcription	119
Translation	312
Folding, sorting and degradation	309
Replication and repair	113
C. Environmental information processing	
Membrane transport	30
Signal transduction	1280
Signalling molecules and interaction	599
D. Cellular processes	
Transport and catabolism	482
Cell growth and death	411
Cellular community—eukaryotes	366
Cell motility	129
E. Organismal systems	
Environmental adaptation	220

^a^List of KEGG pathways used in this study were obtained from Kanehisa laboratory with their kind permission (www.kegg.jp/keg/kegg1.html).

Within the environmental information processing category, the most abundant subcategory was “signal transduction,” with a gene count of 1280. In the cellular processes category, the predominant group was “transport and catabolism,” with a gene count of 482. The genetic information processing category had the largest subgroup called “translation,” with a gene count of 312. In the metabolism category, two categories were more common than others namely “lipid metabolism” (gene count: 260) and “carbohydrate metabolism” (gene count: 208). Last, the organismal systems category had the most abundant group called “environmental adaptation” with a gene count of 222. Furthermore, the Kyoto encyclopedia of genes and genomes (KEGG) annotation statistics are provided in Supplementary Table S11.

### Identification of module and hub genes through protein^_^protein interaction (PPI) network analysis

**Figure 5:**
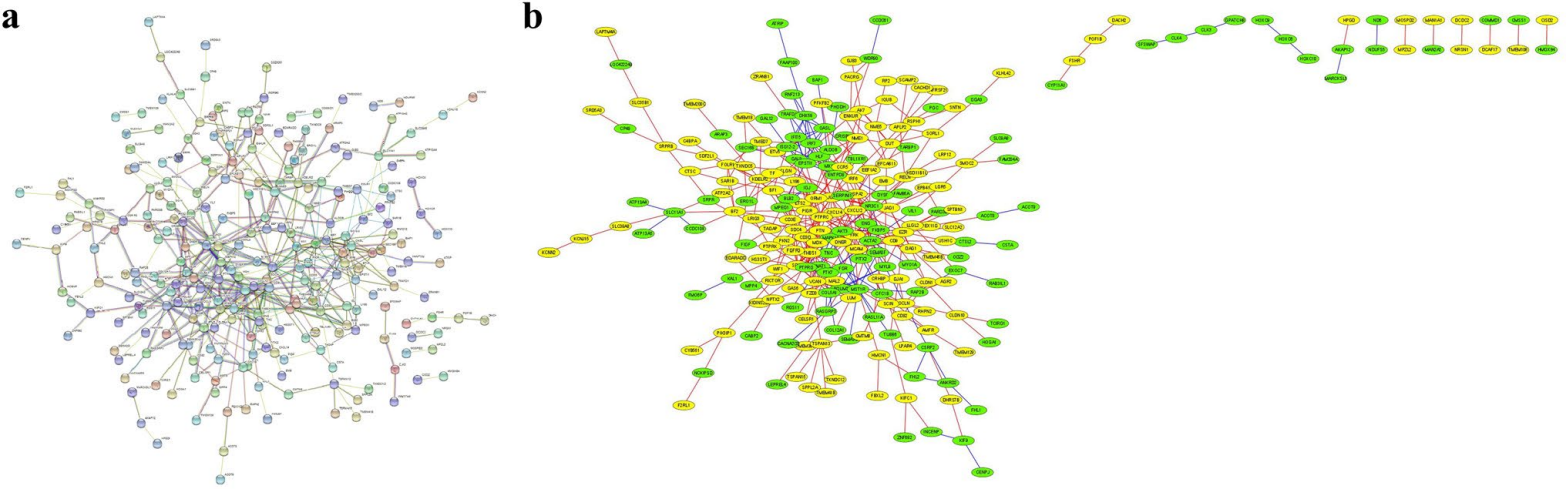
Visualization of protein–protein interaction (PPI) network. (**a**) Protein–protein interactions (PPIs) DEGs were analysed using the STRING database. (**b**) Protein–protein interactions (PPI) were established using Cytoscape software (v3.9.1). The yellow circular nodes represent upregulated genes, whereas the green circular nodes indicate downregulated genes.

According to the analysis of the STRING (Search Tool for the Retrieval of Interacting Genes/Proteins) database, the protein^_^protein interaction (PPI) network of 324 DEGs was observed to have 324 nodes and 439 edges after applying appropriate filters. The expected number of edges was 305, and the average node degree was 2.71, indicating that each node had at least 2.71 interacting nodes. The average local clustering coefficient was 0.373, and the protein^_^protein interaction (PPI) enrichment value was 3.56e^−13^ (Fig. 5a).

To investigate the upregulated and downregulated differentially expressed genes (DEGs), we imported all 324 DEGs into the STRING database and visualized the network using Cytoscape App software. In the resulting network, yellow nodes represented upregulated DEGs, and green nodes represented downregulated DEGs (Fig. 5b).

**Figure 6:**
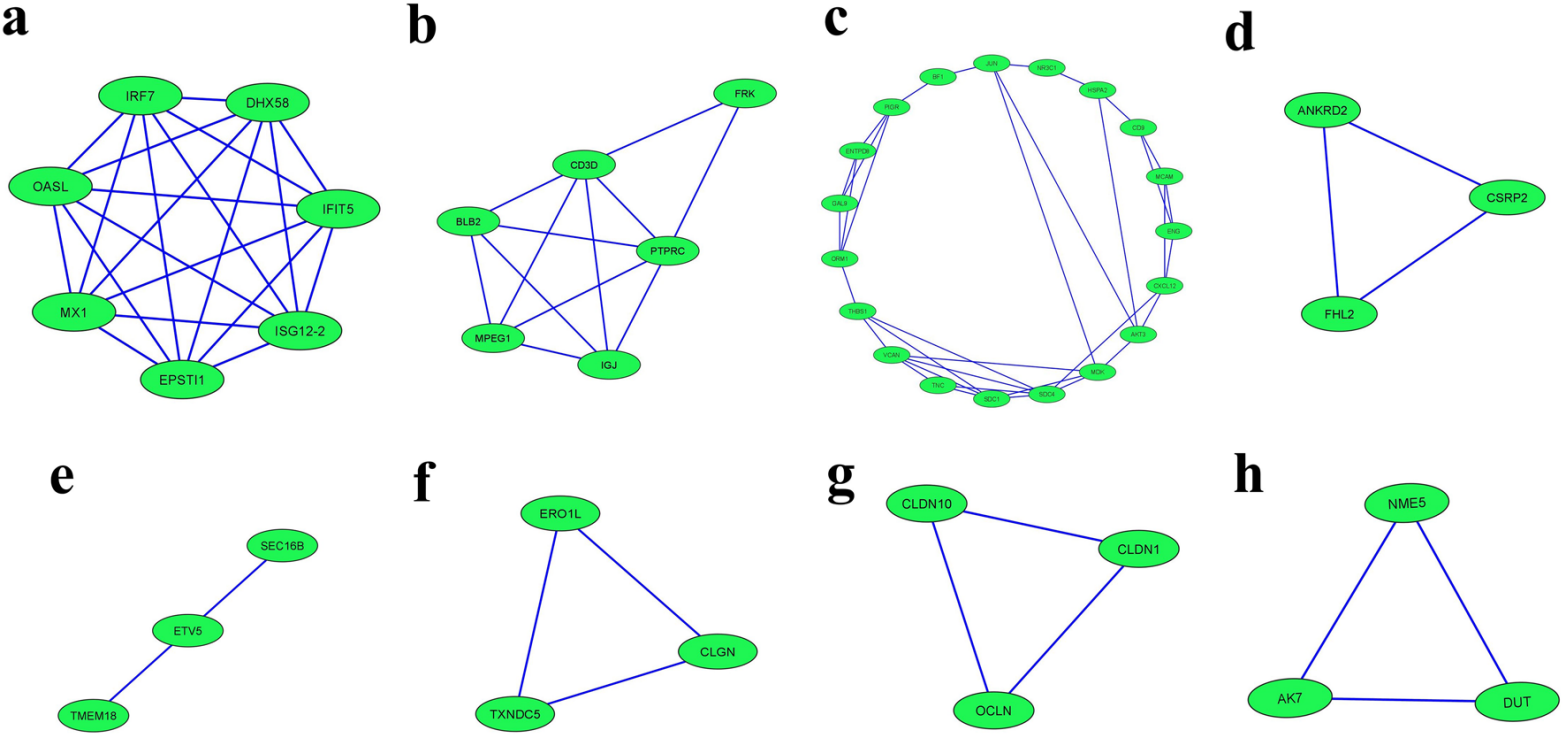
The top eight cluster subnetworks were identified from the protein–protein interaction (PPI) network with the help of Cytoscape App using the MCODE plugin with cluster scores. (**a**) Module 1 (**b**) Module 2 (**c**) Module 3 (**d**) Module 4 (**e**) Module 5 (**f**) Module 6 (**g**) Module 7 and (**h**) Module 8.

To identify functional modules within the network, we performed a functional module analysis using the Molecular Complex Detection (MCODE) plug-in in Cytoscape software. The analysis revealed a total of 8 functional modules (Fig. 6a–h). The top-scoring module was Module 1, which consisted of 7 nodes and 21 edges with a score of 7 (Fig. 6a). Module 2 included 6 nodes and 12 edges with a score of 4.8 (Fig. 6b), and Module 3 consisted of 19 nodes and 35 edges with a score of 3.889 (Fig. 6c). Module 4 included ANKRD2, CSRP2, and FHL2, and had 3 nodes and 3 edges with a score of 3 (Fig. 6d). Module 5 contained SEC16B, ETV5, and TMEM18, and had 3 nodes and 3 edges with a score of 3 (Fig. 6e). Module 6 had ERO1L, CLGN, and TXNDC5, and had 3 nodes and 3 edges with a score of 3 (Fig. 6f). Module 7 included CLDN10, CLDN1, and OCLN, and had 3 nodes and 3 edges with a score of 3. Module 8 also had 3 nodes and 3 edges with a score of 3 (Fig. 6g and h). The detailed results of the functional module analysis are provided in Supplementary Tables S12–S20.

**Figure 7:**
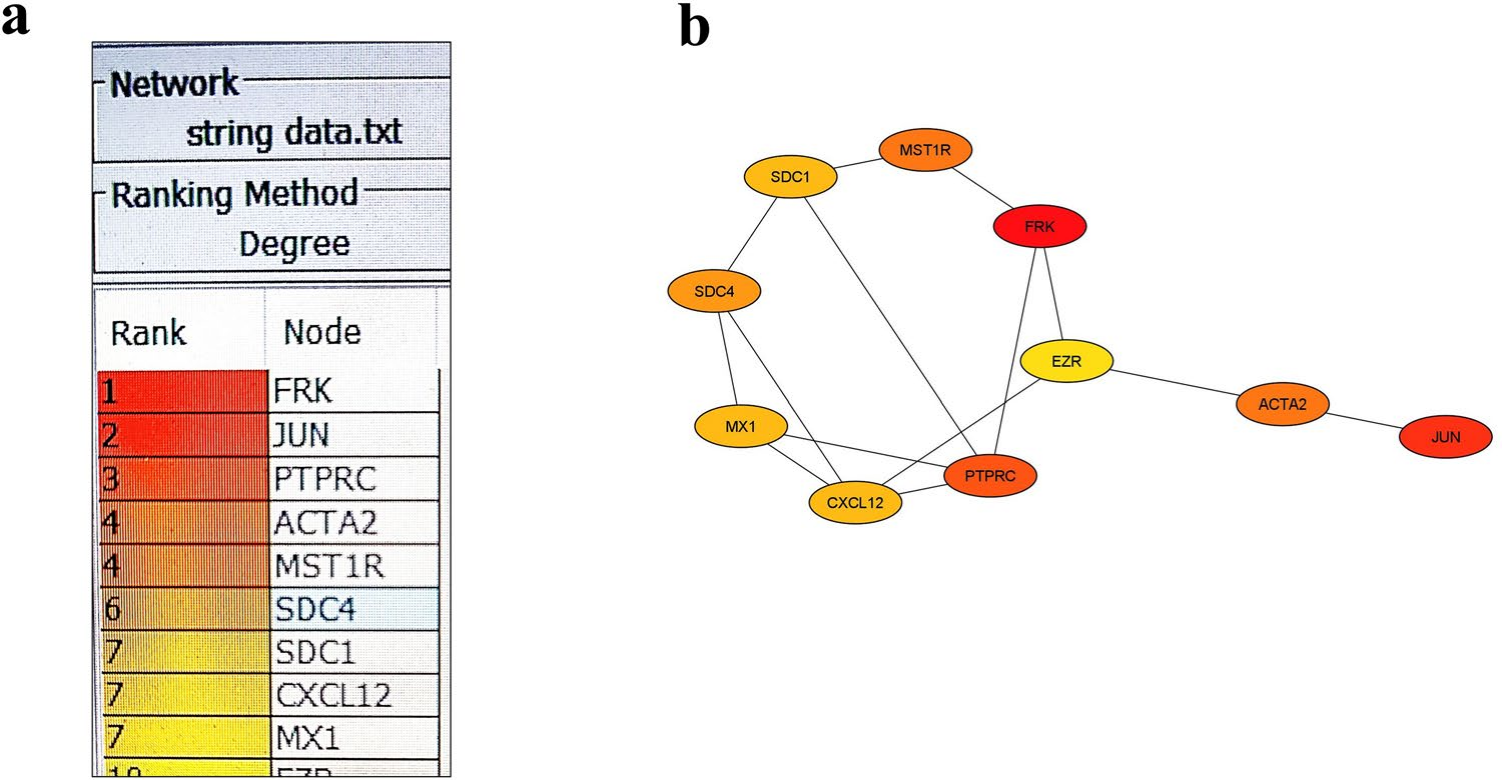
Identification of hub genes from the protein–protein interaction (PPI) network using Cytoscape plugin cytoHubba software. (**a**) Ten hub genes were identified based on their degree algorithm ranking. (**b**) Ten hub genes were identified based on their degree of connectivity.

The hub genes, which are selected based on their high scores in the degree algorithm ranking in CytoHubba (Fig. 7a), are comprised of *FRK* (fyn-related Src family tyrosine kinase), *JUN* (jun proto-oncogene), *PTPRC* (protein tyrosine phosphatase receptor type C), *ACTA2* (actin alpha 2), *MST1R* (macrophage-stimulating 1 receptor), *SDC4* (syndecan 4), *SDC1* (syndecan 1), *CXCL12*, *MX1* (MX dynamin like GTPase 1), and EZR (ezrin), as shown in Fig. 7b.

### Identification of cancer-related gene networks using hub genes

**Figure 8:**
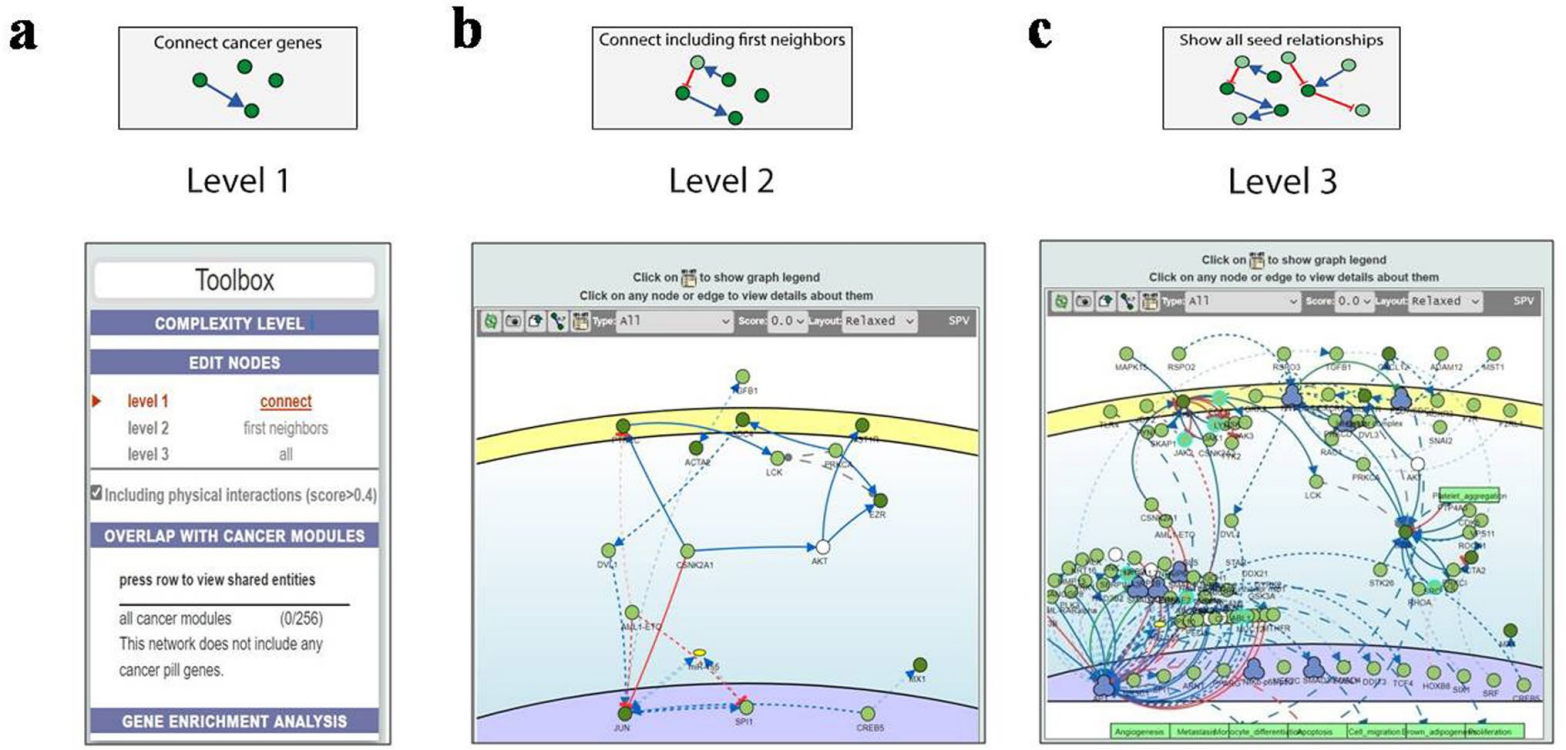
Enrichment of hub genes by connecting the cancer gene network resulted in three graphs. (**a**) At level 1 only direct connections between query hub genes with included physical interactions (score > 0.4) are shown. (**b**) At level 2, genes can be linked by causal interactions with common proteins in the global causal interactome (physical interactions score > 0.4). (**c**) Finally, at level 3, all the interactions of the query cancer hub genes are shown (physical interactions score > 0.4). Paths coloured blue indicate upregulation, red indicate downregulation, green indicate physical interaction, light black indicates unknown, bright black indicates that, large dotted lines indicate indirect interaction, and small dotted lines indicate binding. The image was generated using Signor web-based platform (version 3.0) available at https://signor.uniroma2.it/CancerGeneNet). No additional software was used in the creating this image.

Subsequently, these ten hub genes underwent CancerGeneNet network analysis, resulting in three different types of graphs. In Fig. 8a, a direct connection was observed between the query hub genes at Level 1. Figure 8b shows that the hub cancer genes were connected and interacted with two cancer genes, forming the first neighbours at Level 2. Figure 8c displays the protein-to-protein (PPI) interactions, which were related to external stimuli, including angiogenesis, metastasis, apoptosis, monocyte differentiation, cell migration, brown adipogenesis, and proliferation, at Level 3. Furthermore, Table 4 provides information on the enrichment of hub genes involved in cancer pathways.

**Table 4:** Gene enrichment analysis of hub genes.

Cancer module	Hits	p-values
AML1-ETO in AML	4/18	< 1.0E−4
RTKs in cancer	1/12	< 1.0E−4
ASXL1 in AML	1/13	< 1.0E−4
FLT3 in AML	1/40	< 1.0E−4
Triple mutant AML	1/18	< 1.0E−4
KIT in AML	1/30	< 1.0E−4
AML_TRIPLETS	1/22	< 1.0E−4
Sara	1/67	< 1.0E−4
BCR-ABL in AML	1/20	< 1.0E−4
ErbB receptors in cancer	1/12	< 1.0E−4
WNT/FLT3	3/27	< 1.0E−4
mTOR in cancer	1/15	< 1.0E−4
Onco-fusion protein in AML	1/28	< 1.0E−4
MLL fusion protein in AML	1/21	< 1.0E−4
miRNA in AML	3/35	< 1.0E−4
Wnt in cancer	1/10	< 1.0E−4
FLT3-ITD in AML	3/36	< 1.0E−4
Haematopoiesis transcription control	2/16	< 1.0E−4
NPM1_ new	1/25	< 1.0E−4

## Discussion

Recently, dietary selenium-deficiency has been associated with many diseases in animals and humans^20^. Therefore, we have taken stepwise approaches to verify and support our findings. First, we fed chicks a low-Se diet for 10 weeks, and then, we observed that the Se content in the blood samples was significantly lower in the selenium-deficient group than in the control group. These results indicate that the selenium-deficient model of chicks has been established successfully. Consistent with our findings, previous studies have demonstrated that lower dietary selenium induced severe selenium-deficiency in chicks^21^.

Second, we performed gene expression profiling using RNA-Seq of oviduct samples from the control and selenium-deficient groups. We identified the maximum number of upregulated DEGs (210) and downregulated DEGs (230). The upregulated genes included *AvBD12* (avian beta-defensin 12)*, ATP2A2* (ATPase sarcoplasmic/endoplasmic reticulum Ca^2+^ transporting 2)*, CXCL-12* (cxc motif chemokine ligand 12)*, POF1B* (premature ovarian failure protein 1B)*, SARAF* (store-operated calcium entry associated regulatory factor)*, SLC12A2* (solute carrier family 12 member 2)*, TRAIL-like* (tumour necrosis factor-related apoptosis inducing ligand)*,* and *TXNDC5* (thioredoxin domain containing 5). Downregulated genes such as *AVBD* (avian beta-defensin 9)*,* and *CABP2* (calcium binding protein 2)*,* were activated under selenium deficiency conditions. These genes are involved in various functions such as chemoattractant for avian immune cells^22^, calcium transporters^14^, chemokine members^23^, premature ovarian failure^24^, store-operated calcium entry associated regulatory factor^25^, solute carrier protein^13^, TNF-related apoptosis inducing ligand-like protein^26^, thioredoxin domains^27^, and calcium binding protein similar to calmodulin^28^.

Third, GO annotation and KEGG analyses were used to further elucidate the biological roles of the DEGs during the dietary selenium-deficiency response. Fourth, we constructed a protein^_^protein interaction (PPI) network using the STRING database and detected 8 functional modules and 10 hub genes using the CytoScape software plugins cytoHubba and MCODE. Finally, enrichment analysis of hub genes was performed using CancerGeneNet software. These findings indicated that the hub genes could be potential prognostic biomarkers or therapeutic targets for selenium-deficiency in animal and human models.

In this study, a total of 324 DEGs, 194 upregulated genes and 140 downregulated genes, were identified. Next, the 194 upregulated DEGs were subjected to GO and KEGG pathway enrichment analyses. The biological process (BP) GO terms showed that genes were related to the regulation of cell motility, cell migration, positive regulation of chemotaxis, regulation of signal transduction, cell communication, signalling, locomotion, and cellular component movement. The cellular component (CC) GO terms showed that the genes were associated with the endoplasmic reticulum, endomembrane system, membrane and vesicle. The molecular function (MF) GO showed that the genes were related to protein-containing complex binding and protein binding activity. These ontologies are represented by genes involved in the impaired immune system and some genes related to tumorigenesis. Previous studies showed that selenium modulates intracellular signalling and cell growth^29^.

Furthermore, significant DEGs between the control and selenium-deficient samples were mapped to reference canonical pathways in the KEGG database. A total of 10,266 of the control and selenium-deficient groups were categorized into 23 major KEGG pathway and were classified into five larger pathway categories: metabolism, genetic information processing, environmental information processing, cellular processes, and organismal systems (Table 3). The highest number of gene-associated categories was assigned to the environmental information processing category, followed by metabolism, cellular process, and genetic information processing, whereas the lowest gene count was related to the organismal systems category. In terms of signal transduction, many pathways such as *MAPK* (mitogen-activated protein kinase)*, ErbB* (erythroblastic oncogene B)*, Ras* (rat sarcoma virus)*, Wnt* (wingless-related integration site)*, Rap1* (ras-related protein 1), *Notch, TGF-beta* (transforming growth factor beta)*, Hippo, Apelin, NF-kappa B* (nuclear factor-kB)*, PI3K-Akt* (phosphoinositide-3-kinase-protein kinase B/Ak strain transforming)*, cAMP* (cyclic adenosine monophosphate)*,* and *phospholipase D* indicate a large amount of signal generation during selenium deficiency conditions. Earlier studies revealed that selenium deficiency caused increased cancer risk^30^.

In our study, we identified hub genes according to the degree algorithm score in cytoHubba. The top ten highest-scored genes were selected as the hub genes: *FRK* (fyn-related src family tyrosine kinase), *JUN* (jun proto-oncogene), PTPRC (protein tyrosine phosphatase receptor type C), *ACTAC2* (smooth muscle actin alpha 2), *MST1R* (macrophage-stimulating 1 receptor), *SDC4* (syndecan 4), *SDC1* (syndecan 1), *CXCL12* (c-x-c motif chemokine ligand 12), *MX1* (MX dynamin like GTPase 1) and *EZR* (ezrin). FRK, the top hub gene, encodes a Fyn-related Src family tyrosine kinase^31^. Previous studies have reported that the FRK gene plays an important role during oncogenesis and progression and is regulated in a tissue specific manner^31^.

The second hub gene was JUN, which and belongs to the basic region leucine zipper family, and constitutes activator protein-1 (AP-1) thereby regulating transcription and cellular activities such as proliferation, tissue morphogenesis, apoptosis, and tumorigenesis^32^. PTPRC (protein tyrosine phosphatase receptor type C), the third hub gene, encodes a member of the protein tyrosine phosphatase (PTP) family^33^. PTPRC is also known as CD45 and it functions as a signalling gatekeeper in T cell^34^. It has been reported that PTPRC is related to multiple kinds of malignancies, such as gastric cancer and breast cancer^33^

The ACTA2 gene encodes smooth muscle specific α-actin, a component of the contractile apparatus of vascular smooth muscle cells^34^. Lee reported that ACTA2, which is involved in mechanical tension, cell movement and shape resulted in dynamics of cytoskeletal structures for invasion and metastasis in tumors^35^. The MST1R gene encodes a protein that functions as a tyrosine kinase receptor for macrophage-stimulating protein^36^. Wang reported that MST1R plays an important role in host defence including tissue pairing and inhibition of inflammation induced by pathogens^37^. Sakamoto et al.^38^ showed that higher MST1R expression led to increased ciliary motility which prevented chronic infection.

SDC4 (syndecan-4) is an important hub gene. It is ubiquitously expressed, and a transmembrane proteoglycan bearing heparan sulfate chains^39^. Keller-Pinter suggested that SDC4 (syndecan-4), plays several roles in growth factor binding, small GTPase Rac1 activity, intracellular calcium-level regulation, extracellular matrix component and focal adhesion kinase phosphorylation regulation^40^. SDC4 (syndecan-4) impaired function causes the development of breast cancer, prostate cancer and many other cancers^41,42^. The SDC1 gene (encodes the protein of syndecan-1) is an evolutionarily conserved type I transmembrane protein family and lack a common molecular structure^43^. Several studies have shown that the expression of syndecan-1 is different in different cancer types^44^.

The CXCL12 gene, also known as stromal cell-derived factor-1 (SDF-1) is a crucial chemokine that is involved in embryogenesis, haematopoiesis, lymphopoiesis, angiogenesis, and inflammation^45^. Previous studies have shown that CXCL12 is related to various cancers, including pancreatic cancer, colorectal cancer, breast cancer, and cervical cancer^46–48^. Myxovirus resistance protein 1 (MX1) is a GTPase that inhibits the multiplication replication of many RNA viruses and is a downstream target for the type I IFN pathway of the innate response^49^. Racicot found that MX1 (Myxovirus resistance protein 1) is an essential component of exosomes secreted by uterine epithelial cells in avian^50^. Another study by Feng et al.^51^ suggested that Myxovirus resistance protein 1 is an important marker for organ damage.

EZR (encodes the ezrin protein) is another important hub gene and is a member of the ezrin-radixin-moesin (ERM) protein family^52^. Li, reported that ezrin is mainly expressed on top of the cell surface and maintains the polarity of epithelial cells^53^. Recent studies have found that ezrin participates in the invasion and metastasis of cancer cells^54^.

Furthermore, we used the CancerGeneNet network tool^55^, and we identified that enrichment analysis of the cancer module yielded more hits to AML1-ETO in AML (acute myeloid leukaemia). These studies demonstrate that these 10 hub genes are correlated with different cancers and are consistent with our results, which predicted that they have the potential to become cancer biomarkers. Hence, the top 10 hub genes were related to cancer pathways.

In summary, we first compared the expression of all genes between selenium-deficient and control oviduct samples of chicks by RNA-Seq and found significant changes in selenium status linked with various genes involved in selenium-deficiency. Subsequently, we also performed integrative bioinformatic analysis to identify the key genes, and potential pathways involved in the dietary selenium response. In this context, *FRK* (fyn-related Src family tyrosine kinase)*, JUN* (jun proto-oncogene)*, PTPRC* (protein tyrosine phosphatase receptor type C)*, ACTAC2* (smooth muscle actin alpha 2)*, MST1R* (macrophage-stimulating 1 receptor)*, SDC4* (syndecan 4)*, SDC1* (syndecan 1)*, CXCL12* (c-x-c motif chemokine ligand 12)*, MX1* (MX dynamin like GTPase 1) and *EZR* (ezrin) were identified and verified as hub genes. These hub genes are related to different cancer pathways. Hence, our study provides a strong basis for future cancer and selenium-deficiency targeted therapies, and these 10 hub genes could potentially be new selenium-deficiency gene targeted therapies. Further studies are needed to confirm our findings to determine the exact mechanism behind selenium deficiency in normal humans and their related complications.

## Methods

### Statement

The study followed the guidelines of ARRIVE.

### Birds

A total of thirty-six, 16-week-old white leghorn chickens with an average initial weight of 55.8 g were used. All birds were purchased from Shree Sai Krishna Commercial Poultry Farm, Namakkal, Tamil Nadu, India.

### Experimental design

Chickens were randomly allotted into two dietary treatment groups, the control (basal diet) and selenium-deficient diet groups. Each group consisted of 6 birds with three replicates in 18 different cages (two birds per cage). Each cage was equipped with one nipple cup, and one feeder and the cage size was H40 × W40 × D40 cm. All birds were offered free access to water and an experimental diet ad libitum*,* followed by a 14 L:10 D lighting schedule at an intensity of 40 lx. The common infectious diseases were prevented prophylactically by administering Clostin sulphate, Neomycin sulphate and spectinomycin in the drinking water^56^. Throughout the study, daily observations were conducted to record the overall health of the subjects, as well as any clinical symptoms related to selenium deficiency diseases and mortality and detailed incidents of ED based on gross appearance^57^. All birds are safeguarded against viral infection administrating the Newcastle disease virus (NDV) vaccine through both the intraocular and intranasal routes^58^.

### Establishment of a dietary selenium-deficient chick model

A basal diet was formulated for chicks, utilizing corn-soya bean meal and adhering to the National Research Council (NRC, 1994) guidelines^59^ for their nutritional requirements, as demonstrated in Table 5. The chicks in the control group were fed with basal diet alone for 10 weeks, which contained 0.012 mg/kg of selenium. In contrast, the chicks in the selenium deficient group were given a diet with a selenium content of only 0.002 mg/kg for 10 weeks, with the purpose of inducing state of selenium deficiency in this group, as illustrated in Table 6.

**Table 5:** Composition of basal diet.

Ingredients	Percentage
Corn	63
Soybean meal	21
Limestone	7
Bran	5
Vitamin-mineral premix^a^	4
Nutrient level	
Crude Protein %	16.5
Crude Fibre %	3.35
Lysine %	0.7
Methionine %	0.4
Cystine %	0.3
Digestible energy (ME)/kJ/kg	11.4
Phosphorus %	0.7
Calcium %	3.5
^b^Selenium per mg/Kg	0.012

^a^Vitamin-mineral premix (per Kg Diet): 26,300 IU vitamin A, 8000 IU vitamin D3, 370 IU vitamin E, 40 mg vitamin K3, 35 mg vitamin B1, 100 mg vitamin B2, 30 mg vitamin B6, 0.6 mg vitamin B12, 50 mg niacin, 12 mg pantothenic acid, 13 mg folic acid, 0.8 mg biotin, 110 mg choline chloride, 50 mg Fe, 1.5 mg Cu, 50 mg Mn, 12 Zn, 0.095 mg I.

^b^The analyzed value was the selenium content in the basal diet.

**Table 6:** Composition of selenium-deficient diet.

Ingredients	Percentage
Corn	64
Soybean meal	21
Limestone	7
Bran	5
Vitamin-mineral premix^a^	3
Nutrient level	
CP %	16
Crude Fibre %	4
Lysine %	0.8
Methionine %	0.4
Cystine %	0.5
Digestible energy (ME)/kJ/kg	12
Phosphorus %	0.6
Calcium %	3.5
^ b^Selenium per mg/Kg	0.002

^a^Vitamin-mineral premix (per Kg Diet): 24,500 IU vitamin A, 6000 IU vitamin D3, 570 IU vitamin E, 30 mg vitamin K3, 45 mg vitamin B1, 111 mg vitamin B2, 30 mg vitamin B6, 0.5 mg vitamin B12, 40 mg niacin, 15 mg pantothenic acid, 12 mg folic acid, 0.6 mg biotin, 100 mg choline chloride, 30 mg Fe, 1.6 mg Cu, 30 mg Mn, 15 Zn, 0.090 mg I.

^b^The selenium content in a selenium-deficient diet was the analyzed value.

### Ethical statement

The experimental protocols in this study involving animals were carried out according to the guidelines of the Committee for the Purpose of Control and Supervision of Experiments on Animals (CPCSEA), New Delhi, India^60^ and approved by the Institutional Animal Ethical Committee of Jeeva Life Science, Hyderabad, Telangana, India (Ethical Committee Approval No: CPCSEA/IAEC/JLS/13/08/2014; dated 21.8.20). This study was performed in accordance with ARRIVE guidelines.

### Statistical analysis

The statistical analysis was performed by IBM SPSS 20.0 Software (SPSS Inc., Chicago, IL, United States). Statistical significance between groups was determined by one-way ANOVA followed by a post hoc Tukey HSD comparison test to identify differences in means among dietary treatments for selenium concentration in blood samples. *Asterisk indicates statistical significance (*p* < 0.05).

### Sample collection

Blood samples were taken from the main wing vein and collected into an anticoagulant tube after 10 weeks during the whole feeding period. Plasma was separated by centrifugation at 4 °C, and 3000 rpm for 10 min and stored at − 30 °C for further analysis. After dietary treatments for 10 weeks, 3 randomly chosen chickens from each group were slaughtered 15–20 h post-ovulation. The oviduct was sampled and frozen in liquid nitrogen immediately. All samples were stored at − 80 °C prior to further analysis for RNA-Seq.

### Determination of selenium content

The selenium content in the diet and blood samples was measured according to Pan et al.^61^ with an atomic fluorescence spectrometer (AF-610A, Beijing Beifen-Ruili Analytical Instrument, Yangzhou, China).

### Total RNA isolation and qualitative and quantitative analysis

Total RNA was isolated from the oviduct samples using a commercially available Quick-RNA Miniprep Plus kit (ZYMO Research) according to the manufacturer’s instructions. The quality and quantity of the isolated RNA samples were checked on 1% denaturing RNA agarose gel and a NanoDrop, respectively.

### Illumina NextSeq500 PE library preparation

The RNA-Seq paired end sequencing libraries were prepared from the QC passed RNA samples using an Illumina TruSeq Strand mRNA sample prep kit. Briefly, mRNA was enriched from the total RNA using poly-T attached magnetic beads, followed by enzymatic fragmentation, 1st strand cDNA conversion using SuperScript II and Act-D mix to facilitate RNA dependent synthesis. The 1st strand cDNA was then synthesized to the second strand using a second strand mix. The double cDNA was then purified using AMPure XP beads followed by A-tailing, and adapter ligation and then enriched by limiting the number of PCR cycles.

### Quantity and quality check (QC) of library on Agilent 4200 Tape Station

The PCR enriched libraries were analysed on a 4200 Tape Station system (Agilent Technologies) using high sensitivity D1000 Screen Tape as per the manufacturer’s instructions.

### Cluster generation and sequencing

After we obtained the Qubit concentration for the libraries and the mean peak sizes from the Agilent Tape Station profile, the PE Illumina libraries were loaded onto NextSeq500 for cluster generation and sequencing. Paired-End sequencing allows the template fragments to be sequenced in both the forward and reverse directions on NextSeq500. The kit reagents were used in the binding of samples to complementary adapter oligos on paired-end flow cells. The adapters were designed to allow selective cleavage of the forward strands after resynthesis of the reverse strand during sequencing. The copied reverse strand was used to sequence from the opposite end of the fragment.

### High quality read statistics

The sequenced raw data of the control and selenium-deficient groups were processed to obtain high quality clean reads using Trimmomatic version 0.38 to remove adapter sequences, ambiguous reads (reads with unknown nucleotides “N” > 5%), and low-quality sequences (reads with more than 10% quality threshold (QV) less than 20 phred score). Furthermore, a minimum length of 100 nt (nucleotide) after trimming was added. High-quality reads were obtained after removing the adapter and low-quality sequences from the raw data. This high quality (QV less than 20), paired-end reads were used for reference^_^based read mapping. The parameters considered for filtration are as follows: (1) SLIDINGWINDOW: Sliding window trimming of 10 bp, cutting once the average quality within the window falls below a threshold of 20. (2) LEADING: Cut bases off the start of a read, if below a threshold quality of twenty. (3) TRAILING: Cut bases off the end of a read, if below a threshold quality of twenty.

### Reference genome information

The reference genome of *G. gallus* GRCg6a with a genome size of ~ 1.2 GB and the associated annotations were downloaded from Ensembl (ftp://ftp.ensembl.org/pub/release-94/fasta/gallus_gallus/dna/Gallus_gallus.Gallus_gallus-5.0.dna.toplevel.fa.gz).

### Read mapping

The high-quality reads of the control and selenium-deficient samples were mapped to the reference genome of *G. gallus* GRCg6a, mentioned above using TopHatv2.1.1 with default parameters^62^.

### Differential gene expression (DGE) analysis

The Cufflinks version 2.2.1 program assembles transcriptomes from RNA-Seq data and quantifies their expression^63^. The individual gtf files of the transcriptomes were used for differential gene expression (DGE) analysis using cuffdiff software. There are a total of 24,632 protein coding genes present in the annotation file of *G. gallus* GRCg6a. Differential gene expression analysis was performed using Cuffdiff version 2.2.1 between the control and selenium-deficient samples. FPKM values were used to calculate the log fold change as log_2_ (FPKM_Selenium deficient Group/FPKM_Control). Log_2_ fold change (FC) values greater than zero were represented as upregulated. However, log_2_-fold change (FC) values less than zero were considered downregulated, and a *p* value threshold of 0.05 was used for statistically significant results.

### Heatmap

An average linkage hierarchical cluster analysis was performed on the top 50 differentially expressed genes (DEGs), of the control versus selenium-deficient group combination using a multiple experiments viewer (MeV v4.9.0)^64^. The heatmap shows the level of gene abundance. Levels of expression are indicated as the log_2_ ratio of gene abundance between control and selenium-deficient samples. Differentially expressed genes (DEGs) were analysed by hierarchical clustering. In this regard, heatmaps were constructed using the log transformed and normalized value of genes according the Pearson uncentred distance and average linkage method. In the heatmaps, each horizontal line represents a gene. The colour denotes the logarithmic intensity of the expressed genes; relatively high expression values are shown in red colour and lower expression is shown in green.

### Scatter plot

The Eurofins proprietary R script was used to graphically depict the expression of genes in two distinct conditions of each sample combination i.e., control and selenium-deficient groups. It helps to identify genes that are differentially expressed in one sample with respect to another and allows comparing two values associated with genes. In the scatter plot, each dot denotes a gene. The vertical position of each gene represents its expression level in the control samples while the horizontal position represents its expression level in the selenium-deficient group. Moreover, genes that fall above the diagonal are indicated to be overexpressed. Conversely, genes that fall below the diagonal are denoted as underexpressed as compared to their median expression level in the experimental grouping of the experiment.

### Volcano plot

The Eurofins proprietary R script was used to depict the graphical representation and distribution of differentially expressed genes that were found in control as well as selenium-deficient diet groups. The ‘volcano plot’ displayed expressed genes along dimensions of biological as well as statistical significance. The red colour block on the right side of zero represents the upregulated genes whereas the green colour block on the left side of zero represents significant downregulated genes. The Y-axis denotes the negative log of the *p* value of the performed statistical test where data points with low *p* values are represented as highly significant and appear towards the top of the plot. The grey colour block shows the nondifferentially expressed genes.

### Gene Ontology (GO) and pathway analysis

Gene Ontology was performed by using g: Profile, a public web server (http://biit.cs.ut.ee/gprofiler/), and the parameters for the enrichment analysis were as follows. A specific organism was chosen “*Gallus gallus*”. GO analyses (GO: BP (biological process); GO: CC (cellular component); GO: MF (molecular function)^19^. The functional annotations of genes were used against the curated KEGG (Kyoto Encyclopedia of Genes and Genomes) GENES database using KAAS (KEGG Automatic Annotation Server-. (http://www.genome.jp/kegg/ko.html)^65^. The KEGG Orthology database of the “*Gallus*” family was used as the reference for pathway mapping. The result contains KEGG Orthology (KO) assignments and automatically generated KEGG pathways using the KAAS BBH bidirectional best hit (BBH) method against the available database.

### Protein^_^protein interaction (PPI) network construction and module analysis

The STRING (Search Tool for the Retrieval of interacting Genes/Proteins) database (https://string-db.org/) is one of the largest databases that searches for protein^_^protein interactions^66^. We selected “Multiple proteins” in the left column and entered gene names in the right column, and then picked the organism as “*Gallus gallus*”, chose the minimum-required interaction score as “medium confidence as 0.400” and fixed disconnected nodes in the network, clicked the “Export” option, downloaded the file in TSV format (Supplementary Table S21) and imported it into Cytoscape software (version 3.9.1; https://cytoscape.org/) which is a very powerful tool for visualizing network data^67^. Then, a plug-in in Cytoscape MCODE (molecular complex detection) version 1.5.1^68^ was used to cluster the protein network to build functional modules. The default parameters were degree cut-off = 2, node density cut-off = 0.1, node score cut-off = 0.2, K-core = 2 and max. depth = 100.

### Identification of enrichment analysis of hub genes

The hub genes of the PPI network of DEGs were evaluated by using the cytoHubba (version 1.6) plugin in CytoScape (version 3.9.1) to identify the top 10 hub genes according to the degree algorithm^69^. Enrichment analysis of hub genes by the CancerGeneNet database is a freely available resource with graphical representations of directed interactions between proteins (https://signor.uniroma2.it/CancerGeneNet/)^70^.

## Supplementary Information

Supplementary Tables.

Supplementary Tables.

Supplementary Table S5.

Supplementary Tables.

Supplementary Table S8.

Supplementary Tables.

Supplementary Table S11.

Supplementary Tables.

Supplementary Table S21.

## Data Availability

The datasets generated during and/or analysed during the current study are available in the Fig share links provided in the table below.

**Table:** 

Items	Brief description	Figshare links
Table S1 & S2	High-quality read statistics; Mapping statistics of groups	10.6084/m9.figshare.21702191
Table S3 & S4	List of upregulated differentially expressed genes (DEGs) & List of downregulated differentially expressed genes (DEGs)	10.6084/m9.figshare.21702227
Table S5	Differentially genes expression (DGEs) summary	10.6084/m9.figshare.21702278
Table S6 & S7	Raw data of all genes: List of differentially expressed genes (DEGs)	10.6084/m9.figshare.21702302
Table S8	List of significant differentially expressed genes (DEGs)	10.6084/m9.figshare.21702452
Table S9 &S10	List of genes in exclusive control group; List of genes in exclusive selenium deficient group	10.6084/m9.figshare.21702455
Table S11	Summary of KEGGs pathways annotation statistics	10.6084/m9.figshare.21702464
Table S12, S13, S14, S15, S16, S17, S18, S19 & S20	Cluster genes network analysis (cluster genes 1–8)	10.6084/m9.figshare.21702485
Table S21	STRING database file to construct hub genes	10.6084/m9.figshare.21702491
